# Overexpression of RAD51B predicts a preferable prognosis for non-small cell lung cancer patients

**DOI:** 10.18632/oncotarget.20676

**Published:** 2017-09-06

**Authors:** Mengyin Wu, Zufeng Sheng, Lingyan Jiang, Zhengyuan Liu, Yuhua Bi, Yueping Shen

**Affiliations:** ^1^ Department of Epidemiology and Biostatistics, School of Public Health, Medical College, Soochow University, Suzhou, China

**Keywords:** *RAD51B*, non-small-cell lung cancer, homologous recombination repair, TCGA, prognosis

## Abstract

Lung cancer is the leading cause of cancer-related death. The majority of patients are diagnosed at an incurable advanced stage with poor prognosis. A recent study associated the methylation of homologous recombination genes with expression of immune checkpoints in lung squamous cell carcinoma. However, the correlation between them remains unclear. In our study, we propose that RAD51B, a repair gene in the homologous recombination process, which is noticed to be a key player in the maintenance of chromosome integrity and in sensing DNA damage, can act as an independent factor affecting the prognosis of non-small-cell lung cancer (NSCLC). Univariate analysis showed that overexpression of RAD51B is statistically significant correlated with better prognosis (*P*=0.013). Further, the multivariate Cox regression analysis showed that the morbidity of patients with high expression of RAD51B was decreased by 26% compared to those with low expression (HR=0.74, 95%CI: 0.59-0.93), especially for the patients with squamous cell carcinoma (HR=0.68, 95%CI: 0.51-0.90). In conclusion, RAD51B in mRNA level can be an important indicator to decide the prognosis of NSCLC and its overexpression predicts a preferable prognosis for NSCLC. Our results serve as a foundation for the investigation of the role of RAD51B in NSCLC, which may lead to potential therapeutic innovations.

## INTRODUCTION

Lung cancer remains the most frequently diagnosed cancer worldwide and the leading cause of cancer-related deaths. Over half of patients die within one year after being diagnosed with lung cancer and the 5-year survival rate is only about 17.8% [1]. Therein, non-small-cell lung carcinoma (NSCLC) accounts for approximately 85% of all these cases. Due to its characteristics of high recurrence and metastasis after surgery, the clinical prognosis is unfavorable, particular if diagnosed at a later stage. Hence, finding significant, early-presented, prognosis factors of NSCLC can aid prompt treatment. However, the clinical prognosis factors currently available only exhibit in a small fraction of NSCLC deaths. Like PD-1 inhibitor, as a powerful drug to unleash the immune system, which used to hold the promise of wiping out cancer for some people with advanced lung cancer, but two recent studies suggest that it might backfire in some patients - speeding cancer’s spread [2, 3]. Therefore, there is a need to explore new biomarkers that correlate with morbidity of NSCLC patients. Furthermore, targetable mutations and targeted therapy resistance development in 50% of NSCLC cases emphasizes the significance for developing new prognostic indicators and alternative therapeutic strategies for treating NSCLC [4].

The accumulation of progressive damage to nuclear DNA is considered to be a prominent factor in age-associated diseases [5]. Notably, DNA double-strand breaks (DSBs) are particularly detrimental which can result in mutations and chromosomal translocations and may induce cancer [6]. Therefore, they must be efficiently repaired to preserve genome integrity and functionality. One of the major repair pathways is homologous recombination, which works as an error-free mechanism by employing the homologous sequence in the sister chromatid as a template to prime repair synthesis and restore chromosome integrity [7]. The defining step of homologous strand exchange is directed by the RAD51 protein [8]. It forms a nucleoprotein filament by polymerizing onto resected DNA ends to promote this exchange process [9]. Previous researches have shown that the aberration of its expression has significant effects on tumorigenesis and tumor progression.

It has also been suggested that RAD51 homologues (RAD51B, RAD51C, RAD51D, XRCC2, and XRCC3) are important cofactors for RAD51 protein in the process of chain transfer or chain interaction to initiate DNA homologous pairing [10]. They share 20-30% DNA sequence, and have been identified in vertebrates, with animal cells defective in any of these showing spontaneous chromosomal aberrations [11]. In addition to their independent functions, they were observed to form two major complexes: one defined as BCDX2 is made of RAD51B, RAD51C, RAD51D and XRCC2, whereas the other named CX3 consists of RAD51C and XRCC3. In addition, RAD51B-RAD51C (BC) and RAD51D-XRCC2 (DX2) sub-complexes are formed, which act at both early and late stages of the homologous recombination repair process [12]. Notably, the sub-complex BC exhibits single-stranded DNA-dependent ATPase activity involving RAD51 foci formation and reacting to DNA damage, suggesting an early function in the invasion step of homologous recombination [13]. However, previous research mainly revealed the functions performed by RAD51C, the specific activities of RAD51B have not been clarified *in vivo*. Recent reports demonstrated that haploinsufficiency of RAD51B could cause a defect in homologous recombination repair as well as centrosome fragmentation and increased aneuploidy in HCT116 [14]. Moreover, overexpression of RAD51B has been observed to cause cell cycle G1 delay and cell apoptosis, which suggests a significant role in the maintenance of chromosome integrity and in sensing DNA damage [15]. Furthermore, RAD51B gene has been identified as a risk factor for prostate, ovarian, breast, head and neck and other cancer types in recent reports [16–18]. However, no prior research has proposed a link between the expression of RAD51B gene and lung cancer. Also, the study of the RAD51 family still remains poorly understood. Hence, we hypothesize that RAD51 homologues may have a positive correlation with lung cancer prognosis.

In order to verify this hypothesis, we exploited the largest cancer gene information database worldwide, The Cancer Genome Atlas database (TCGA), to screen related genes in mRNA level using Statistical Product and Service Solutions (SPSS23.0) to perform statistical analysis. In our study, we describe a significant result from data analysis, proposing for the first time the function of RAD51B in the prognosis of non-small-cell lung carcinoma patients. Herein, we show that RAD51B overexpression could indicate an increase in the overall survival rate of NSCLC patients, which suggests that RAD51B could act as a new potential biomarker and a predictor of better prognosis of NSCLC patients.

## RESULTS

At the beginning, we analyzed the relationship between the prognosis of NSCLC and RAD51 family mRNA levels that are available from the TCGA database, including RAD51B, RAD51C, RAD51D, XRCC2, and XRCC3, however, no significant correlation was found except for RAD51B (data not shown). Hence, our analysis focuses on RAD51B associated with NSCLC in mRNA level, as follows.

### RAD51B expression level comparison of clinicopathological parameters

In Table 1, we found that there was a higher expression level of RAD51B in NSCLC patients with male, squamous cell carcinoma, EGFR mutation, and no KRAS mutation than the reference groups (all *P*<0.05). However, no significant difference for the expression level of RAD51B was found for age, recurrence, history of smoking, stage and survival outcomes (all *P*>0.05).

**Table 1:** RAD51B expression level comparison of clinicopathological parameters

Clinicopathological parameters		RAD51B expression level					χ^2^/z value	*P* Value
	n	*P*_*25*_	*P*_*50*_(*median*)	*P*_*75*_	*Max*	*Min*		
Gender								
Male	635	5.59	6.05	6.60	9.31	3.54	2.661	**0.008**
Female	427	5.56	5.96	6.38	9.87	3.54		
Age								
≤68	577	5.58	6.00	6.54	9.87	3.56	0.931	0.352
>68	485	5.58	5.99	6.46	9.31	3.54		
Recurrence								
Yes	195	5.62	6.09	6.55	8.39	3.54	1.806	0.071
No	556	5.53	5.93	6.42	9.87	3.75		
^*^Missing	311							
History of smoking								
Non-smoker	93	5.60	6.06	6.45	8.61	4.25		
Reformed smoker	678	5.59	5.99	6.54	9.31	3.54	1.161	0.560
Current smoker	264	5.50	5.99	6.45	9.87	3.56		
Missing	27							
Stage								
Stage I	547	5.59	5.97	6.46	9.31	3.54		
Stage II	294	5.52	6.01	6.50	9.87	3.56	1.687	0.430
Stage III-IV	216	5.65	6.06	6.61	8.14	3.54		
Missing	5							
Pathological type								
Adenocarcinoma^#^	503	5.49	5.90	6.30	9.87	3.54	41.040	**<0.001**
Squamous cell carcinoma^%^	520	5.68	6.17	6.70	9.31	3.56		
Others^&^	39	5.48	5.81	6.25	4.61	7.68		
EGFR mutation								
No	219	5.56	5.91	6.32	8.39	3.54	3.700	**<0.001**
Yes	38	5.88	6.37	6.84	7.98	4.89		
Missing	805							
KRAS mutation								
No	183	5.68	6.09	6.46	8.39	3.54	2.443	**0.015**
Yes	74	5.45	5.83	6.13	7.68	3.77		
Missing	805							
Outcome								
Living	688	5.55	5.99	6.49	9.87	3.54	0.150	0.881
Death	374	5.63	6.01	6.48	8.39	3.54		

# Adenocarcinoma: lung acinar adenocarcinoma, lung adenocarcinoma mixed subtype, lung adenocarcinoma-not otherwise specified (NOS), lung clear cell adenocarcinoma, lung micro papillary adenocarcinoma, lung mucinous adenocarcinoma, lung papillary adenocarcinoma, lung signet ring adenocarcinoma, lung solid pattern predominant adenocarcinoma.

% squamous cell carcinoma: lung basaloid squamous cell carcinoma, lung clear cell squamous cell carcinoma, lung papillary squamous cell caricnoma, lung small cell squamous cell carcinoma, lung squamous cell carcinoma-not otherwise specified (NOS).

&Others: lung bronchiole-alveolar carcinoma mucinous, lung bronchiole-alveolar carcinoma nonmucinous, mucinous (colloid) carcinoma.

### Correlations between clinicopathological parameters and survival time in NSCLC patients

Table 2 describes the OS for 1-year, 2-year and 5-year, the median time with 95%CI, and compares the differences of the survival curves with clinicopathological parameters. In 1062 NSCLC cases, 1-year, 2-year and 5-year OS (95% CI) were 84.24% (81.83%∼86.65%), 70.58% (67.25%∼73.91%) and 41.63% (36.77%∼46.44%), respectively.

**Table 2:** Correlations between clinicopathological parameters and survival time in NSCLC patients

Clinicopathological parameters	Cases N	Overall survival (%)			Median survival time (days) and 95%CI	Chi-square value	*P*-value
		1-year	2-year	5-year			
Gender							
Male	635	81.10	66.70	43.24	1423.0 (1194.0∼1713.0)	1.544	0.214
Female	427	88.77	76.30	37.77	1379.0 (1161.0∼1656.0)		
Age							
≤68	577	85.53	73.51	44.17	1485.0 (1344.0∼1912.0)	5.480	**0.019**
>68	485	82.45	67.33	38.74	1235.0 (1057.0∼1622.0)		
Recurrence							
Yes	195	88.07	63.78	28.19	999.0 (864.0∼1235.0)	35.215	**<0.001**
No	556	87.09	78.87	57.22	2393.0 (n.r.^*^)		
Missing^*^	311	-	-	-	-		
History of smoking							
Non-smoker	93	80.94	71.09	26.80	1073.0 (905.0∼1498.0)	1.019	0.601
Reformed smoker	678	85.64	72.19	44.02	1485.0 (1265.0∼1856.0)		
Current smoker	264	81.01	66.37	38.86	1293.0 (1043.0∼1713.0)		
Missing	27	-	-	-	-		
Stage							
Stage I	547	89.77	80.10	52.07	1912.0 (1656.0∼2174.0)	51.810	**<0.001**
Stage II	294	82.15	66.37	35.66	1147.0 (995.0∼1492.0)		
Stage III-IV	216	72.20	52.50	23.51	807.0 (624.0∼1045.0)		
Missing	5	-	-	-	-		
Pathological type							
Adenocarcinoma	503	87.90	73.55	35.60	1293.0 (1194.0∼1498.0)	1.800	0.180
Squamous cell carcinoma	520	80.32	67.13	44.35	1470.0 (1150.0∼1874.0)		
Others	39	-	-	-	-		
RAD51B (total)							
Lower	758	83.12	68.95	37.76	1288.0 (1417.0∼1470.0)	6.148	**0.013**
Higher	304	86.85	74.39	49.30	1790.0 (1346.0∼2133.0)		
RAD51B (Adenocarcinoma)							
Lower	402	86.89	73.00	33.43	1288.0 (1135.0∼1498.0)	0.240	0.6244
Higher	101	91.49	71.16	31.68	1265.0 (879.0∼1790.0)		
RAD51B (Squamous cell carcinoma)							
Lower	324	77.97	63.10	38.02	1154.0 (974.0∼1640.0)		
Higher	196	83.62	72.85	52.63	1933.0 (1346.0∼2803.0)	7.120	**0.0076**
Total	1062	84.24	70.58	41.63	1379.0 (1235.0∼1640.0)		

n.r.^*^=not reached.

It was found that the increasing OS in NSCLC patients was predominantly associated with younger age (*P*=0.019), no recurrence (*P*<0.001), and earlier UICC stage (*P*<0.001), whereas this increase is not correlated with patients’ gender, history of smoking, or pathological type (Table 2).

**Figure 1:**
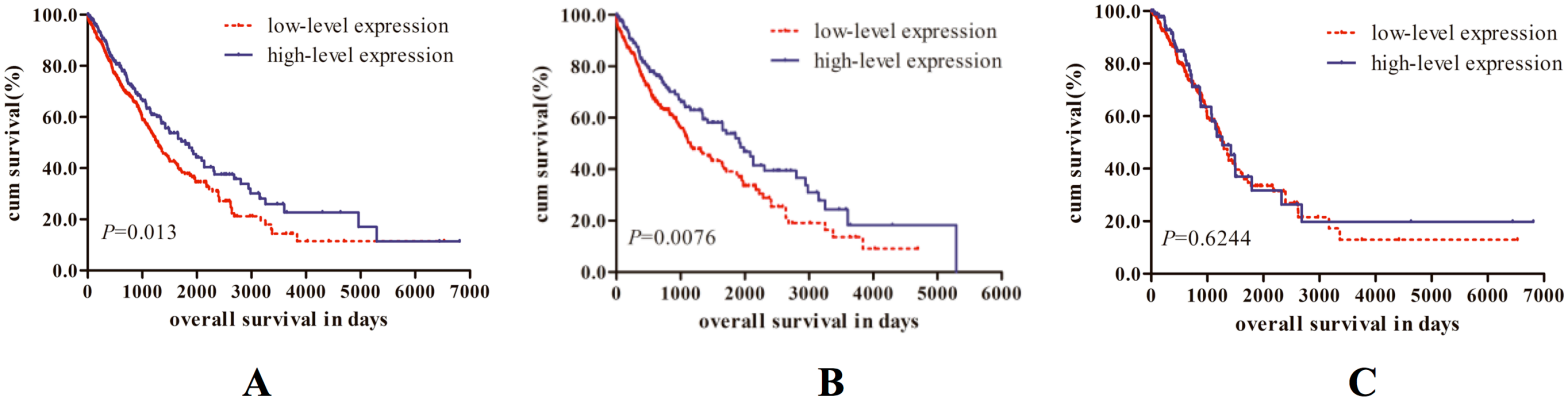
Kaplan-Meier survival curves for patients with high or low level of RAD51B expression in NSCLC **(A)** total patients (n=1062); **(B)** squamous cell carcinoma patients (n=520); **(C)** adenocarcinoma patients (n=503). The *P*-values were computed by log-rank test.

For the total patients, we found that patients with high RAD51B expression have a longer median survival time than those with low RAD51B expression (*P*=0.013, Figure 1A). After stratification analysis, RAD51B overexpression was also found to be associated with the increasing OS in the patients with squamous cell carcinoma (*P*=0.0076, Figure 1B), but this association was not shown in the adenocarcinoma patients (*P*=0.6244, Figure 1C).

### Cox model analysis for the expression of RAD51B and RAD51B’s independent prediction of prognosis in NSCLC patients

According to Table 3, the multivariate Cox analysis for the total patients demonstrates that overexpression of RAD51B is independently associated with better prognosis for NSCLC patients. The HR for death of patients was 0.74 (95%CI: 0.59∼0.93) after adjustment for the potential factors (recurrence, stage, age, gender and history of smoking). In addition, elder age, later UICC stage, and recurrence were significantly associated with the increasing death risk of death for NSCLC patients. In the stratified analysis (Table 4 and Table 5), overexpression of RAD51B was prominently associated with the decreasing death risk of death for patients with squamous cell carcinoma (HR=0.68, 95%CI: 0.51∼0.90). However, a significantly better prognosis effect for RAD51B was not found in NSCLC patients with adenocarcinoma (HR=0.78, 95%CI: 0.53∼1.16).

**Table 3:** Cox model survival analysis of clinicopathological parameters in NSCLC patients (n=1062)

Clinicopathological parameters	HR (95%CI)
RAD51B	
Lower	1.00 (ref)
Higher	0.74 (0.59∼0.93)
Gender	
Female	1.00 (ref)
Male	0.88 (0.70∼1.09)
Age (yrs)	
≤68	1.00 (ref)
>68	1.32 (1.07∼1.63)
Recurrence	
No	1.00 (ref)
Yes	2.01 (1.56∼2.59)
Missing	1.70 (1.32∼2.18)
Smoke history	
Non-smoker	1.00 (ref)
Reformed-smoker	0.85 (0.56∼1.29)
Current-smoker	0.90 (0.58∼1.41)
Missing	0.79 (0.43∼1.48)
Stage	
I	1.00 (ref)
II	1.52 (1.18∼1.95)
III+IV	2.34 (1.83∼3.00)
Missing	n.r.^*^

n.r.^*^ =not reached.

**Table 4:** Cox model survival analysis of clinicopathological parameters in NSCLC patients with squamous cell carcinoma (n=520)

Clinicopathological parameters	HR (95%CI)
RAD51B	
Lower	1.00 (ref)
Higher	0.68 (0.51∼0.90)
Gender	
Female	1.00 (ref)
Male	0.82 (0.59∼1.14)
Age (yrs)	
≤68	1.00 (ref)
>68	1.23 (0.93∼1.63)
Recurrence	
No	1.00 (ref)
Yes	1.74 (1.24∼2.44)
Missing	1.36 (0.97∼1.90)
Smoke history	
Non-smoker	1.00 (ref)
Reformed-smoker	0.38 (0.17∼0.89)
Current-smoker	0.50 (0.21∼1.18)
Missing	0.23 (0.05∼1.15)
Stage	
I	1.00 (ref)
II	1.12 (0.81∼1.54)
III+IV	1.55 (1.09∼2.20)
Missing	n.r.^*^

n.r.^*^=not reached.

**Table 5:** Cox model survival analysis of clinicopathological parameters in NSCLC patients with adenocarcinoma (n=503)

Clinicopathological parameters	HR (95%CI)
RAD51B	
Lower	1.00 (ref)
Higher	0.78 (0.53∼1.16)
Gender	
Female	1.00 (ref)
Male	1.06 (0.76∼1.48)
Age (yrs)	
≤68	1.00 (ref)
>68	1.47 (1.04∼2.07)
Recurrence	
No	1.00 (ref)
Yes	2.60 (1.73∼3.91)
Missing	2.40 (1.60∼3.61)
Smoke history	
Non-smoker	1.00 (ref)
Reformed-smoker	1.09 (0.64∼1.87)
Current-smoker	0.72 (0.39∼1.32)
Missing	0.70 (0.34∼1.45)
Stage	
I	1.00 (ref)
II	2.70 (1.76∼4.14)
III+IV	4.10 (2.78∼6.06)
Missing	n.r.^*^

n.r.^*^=not reached.

## DISCUSSION

The function of RAD51B in cancer cell lines has not been well studied and most previous work has rarely mentioned the association between RAD51B and lung cancer. Herein, we took advantage of TCGA data, screening candidate DNA-repair genes (RAD51B, RAD51C, RAD51D, XRCC2, and XRCC3) with covariance and correlation matrices in mRNA level from complete 1124 complete cases of non-small cell lung cancer clinical data. The Kaplan-Meier analysis results indicate that patients with low level of RAD51B expression exhibited about 6% overall survival rate decreasing compared to patients with high level (*P*=0.013), whereas the remaining genes (RAD51C, RAD51D, XRCC2, and XRCC3) showed no statistical significance (data not shown). This result implies that RAD51B could be a candidate prognostic factor for NSCLC patients. Furthermore, after adjustment for some potential confounding factors, the multivariate Cox regression analysis showed that the death risk of patients with high expressions of RAD51B decreased by 26% compared to those with low expression, especially for NSCLC patients with squamous cell carcinoma. The distinct roles of RAD51B in lung squamous cell carcinoma and lung adenocarcinoma may be contributed to the presence of different signaling pathways or growths factors in these two histopathology types [19]. We are yet to unravel the mechanisms underlying this phenomenon, but the results suggest that RAD51B might be a novel marker, particularly useful for the NSCLC patients with squamous cell carcinoma.

Recently, a few researches have focused on a genetic level to study the association between RAD51B genetic variants and the risk of the male breast cancer in a GWAS study, and the association with death risk of glioblastoma in a case-control study [20, 21]. In accord with our findings, in terms of the epigenetic level, Rieke reported that hypermethylation of RAD51B was associated with an immune-evasive phenotype in squamous cell carcinoma in a recent publication [22], which indicates that DNA methylation-mediated decrease in RAD51B expression levels may predict a poorer prognosis because of activated immune evasion. Nevertheless, we point the effect out directly and our results, based on a population study, provide the first statistical support to RAD51B overexpression leading to an improved prognosis state for NSCLC patients. Furthermore, the finding of Osamu Date shows that haploinsufficiency of RAD51B leads to aberrant homologous recombination repair as well as centrosome fragmentation and increased aneuploidy in HCT116 cells, which indicates that loss of the proper biallelic expression of RAD51B is likely to be linked with malignant transformation by inducing chromosome instability [14]. Also, RAD51B has been shown to interact directly with P53, implying its function as a tumor suppressor [23]. In uterine leiomyoma, the phenomenon of frequent inactivation of RAD51B by translocation between chromosomes 12 and 14 was noticed, supporting a positive role of RAD51B in tumorigenesis [24].

Basic studies on the mechanism of RAD51B were also found to be in accord with our results. Emerging evidence suggests that the highly conserved Saccharomyces cerevisiae RAD51 recombinase plays a key role in eukaryotic, which coats ssDNA ends to assemble a nucleoprotein filament, promoting strand invasion into a homologous duplex to initiate repair synthesis [25, 26]. Moreover, RAD51B repairs various types of DNA lesions and maintains chromosome integrity by promoting the assembly of RAD51 nucleoprotein filaments during this process [27]. Notably, RAD51B has been demonstrated to express at its highest level widely in tissues that are vigorous in recombination work, and RAD51B^-/-^-knockout results in early embryonic lethality and fails to proliferate *in vitro*, which indicates that the RAD51B gene product is essential for cell development and homologous recombination activity [23]. In addition to the functions mentioned above, RAD51B is also proposed to participate in cell cycle control in a direct way. A recent report found that overexpression of the wild-type RAD51B protein of CHO cells containing a mutant P53 can induce G1 delay, which could cause senescence of normal cells but control the proliferation of cancer cells, indicating that hyperexpression of RAD51B may play a positive role in cancer prognosis [15]. In mechanism study, RAD51B protein was found to combine ssDNA and dsDNA in the presence of ATP and Mg2+ or Mn2+ and hydrolyze ATP in a DNA-dependent manner [28], which is critical in processing the Holliday junction, a key intermediate in the homologous recombination repair pathway. Besides, evidence demonstrates that RAD51B could interact with RAD51C to form a highly stable heterodimer, facilitating RAD51 to replace RPA from the nucleo filaments and promoting the DNA strand exchange activity of RAD51-ssDNA filaments [13]. It is noticeable that RAD51C has been proved to be a tumor suppressor [10]. Based on the correlation of structure and biological functions between these two repair genes, there could be a presumption that RAD51B may also function as a non-negligible role in tumorigenesis. Intriguingly, in contrast to our findings on RAD51B, others showed that hyperexpression of RAD51B in a subset of GC (Gastric Cancer) was significantly associated with poor prognosis and resistance to chemotherapy [29]. This apparent discrepancy may be the result of species differences and/or differences between cell types.

In our research, we are facilitated by TCGA highly credible database, which can effectively collect, select, and analyze human tissues for genomic alterations on a very large scale. We thus combined gene information with clinical data, using statistical methods to draw a substantive conclusion. However, since cut-off values and staining patterns remain poorly defined and intratumoral heterogeneity is present, the therapeutic relevance of these biomarkers remains a matter of debate.

Nevertheless, our results provide statistical evidence for RAD51B as an independent factor affecting the prognosis of NSCLC, especially for the patients with squamous cell carcinoma. It also indicates that RAD51B could be a promising site for targeted therapy. To verify this hypothesis, a detailed research of RAD51B in association with its molecular mechanism and clinical trials in NSCLC will be needed. Finally, we hope this research can provide ideas and fundamental theory to support further studies on RAD51B.

## MATERIALS AND METHODS

### Data source

Publicly accessible data from The Cancer Genome Atlas research network (TCGA) was used for determining NSCLC cases (TCGA provisional, 1124 samples) and gene expression (IlluminaHiseq) of lung cancer, from Jul. 2010 to Feb. 2015, downloaded from the Cancer Genomics Browser (http://genome-cancer.ucsc.edu). According to established selection criteria of clinical case parameters, 1062 samples were available for analysis with valid data for RAD51B expression level, event, recurrence, overall survival time, etc. According to the data’s distribution of the RAD51B expression level, we searched for the optimum higher expression cut-off value from *P*_5_ to *P*_95_ respectively, after taking account into both *P*-value and sample size. As a result, we found the *P*_70_ (>6.39) as the optimum cutoff point of RAD51B, which classifies the NSCLC patients significantly after performing the Log-rank test. Thus, we defined patients who carried an RAD51B expression level of >6.39 as the high expression group, with others being the low expression group, resulting in higher and lower expression group samples of 304 and 758, respectively.

### Statistical analysis

We used the Wilcoxon and Kruskal-Wallis rank sum test to test the difference of the expression level of RAD51B between two or more than two groups due to its distribution not following a normal distribution. The overall survival (OS), survival curve, and median survival time were estimated by Kaplan-Meier methods and their difference was compared using the log-rank test. Occurrence of cancer-related death in patients was defined as event failure. The hazard ratio (HR) and its 95% confidence interval (95%CI) of NSCLC for the differentiated death risk and RAD51B expressions were estimated in total subjects or in the stratified groups (adenocarcinoma or squamous cell carcinoma) by the Cox model, after adjustment for the potential factors: recurrence, stage, age, gender and history of smoking. Analyses were performed using SPSS version 23.0 (SPSS Institute Inc. Chicago, IL, USA) with *P*<0.05 considered as statistically significant.

## Abbreviations

NSCLC: Non-small-cell lung cancer
HR: Hazard ratio
DSBs: DNA double-strand breaks
OS: Overall survival
CHO: Chinese hamster ovary
HCT116: Human colorectal cancer cell line
RAD: Radiation-repair gene
XRCC: X-ray repair cross-complementing
